# Courier service for phosphatidylinositol: PITPs deliver on demand

**DOI:** 10.1016/j.bbalip.2021.158985

**Published:** 2021-09

**Authors:** Tim G. Ashlin, Nicholas J. Blunsom, Shamshad Cockcroft

**Affiliations:** Dept. of Neuroscience, Physiology and Pharmacology, Division of Biosciences, University College London, London WC1E 6JJ, UK

**Keywords:** Phospholipase C, Phosphatidic acid, Lipid transfer, Phosphatidylinositol, PITPNC1, Golgi, Membrane traffic

## Abstract

Phosphatidylinositol is the parent lipid for the synthesis of seven phosphorylated inositol lipids and each of them play specific roles in numerous processes including receptor-mediated signalling, actin cytoskeleton dynamics and membrane trafficking. PI synthesis is localised to the endoplasmic reticulum (ER) whilst its phosphorylated derivatives are found in other organelles where the lipid kinases also reside. Phosphorylation of PI to phosphatidylinositol (4,5) bisphosphate (PI(4,5)P_2_) at the plasma membrane and to phosphatidylinositol 4-phosphate (PI4P) at the Golgi are key events in lipid signalling and Golgi function respectively. Here we review a family of proteins, phosphatidylinositol transfer proteins (PITPs), that can mobilise PI from the ER to provide the substrate to the resident kinases for phosphorylation. Recent studies identify specific and overlapping functions for the three soluble PITPs (PITPα, PITPβ and PITPNC1) in phospholipase C signalling, neuronal function, membrane trafficking, viral replication and in cancer metastases.

## 1 Introduction

Phosphoinositides, phosphorylated derivatives of phosphatidylinositol (PI), are key regulators of diverse cellular processes. These include plasma membrane receptor signalling through phospholipase Cs and PI 3-kinases, membrane trafficking, lipid exchange, ion channel regulation and cytoskeletal remodelling. The 3, 4, and 5 positions of the inositol ring of PI are accessible for phosphorylation individually and in combination, resulting in seven distinct phosphoinositides with phosphatidylinositol 4-phosphate (PI4P) and phosphatidylinositol (4,5) bisphosphate (PI(4,5)P_2_) being the most abundant species. The 3-phosphorylated species (PI3P PI(3,4)P_2_, PI(3,5)P_2_ and PI(3,4,5)P_3_), and PI5P are present at substantially lower amounts in comparison. Phosphoinositides are the products of lipid kinases and phosphatases that are present in many subcellular compartments including the Golgi, endosomes and the plasma membrane. However, as the site of PI synthesis is the ER, PI delivery to other membrane compartments is required [1]. The hydrophobic nature of lipids in general means they require specific mechanisms for their distribution to target membranes. Whilst vesicular transport can deliver bulk lipids to target membranes, the more specific mechanism for delivery of specific lipids is accomplished by lipid transport proteins (LTPs). Many different families of LTPs have now been characterised and are widely distributed both intracellularly and extracellularly [2,3]. LTPs are characterised by a lipid binding domain that can encapsulate a single lipid monomer; the domain can exist as a single-domain soluble protein or be part of a multi-domain protein. A major development in the LTP field is the observation that many LTPs deliver lipids at membrane contact sites [4]. For multi-domain proteins, the lipid transfer domain co-exists with membrane-targeting domains that can facilitate the formation of membrane contact sites. Some LTPs may also associate with pre-existing membrane contact sites formed by other tethering factors. Whether single domain LTPs also function at membrane contact sites is not known, and if they do, it remains a major challenge to identify how they are recruited. Until now, LTPs were thought to bind and transfer a single lipid molecule but recent studies have identified proteins that contain a long hydrophobic groove that can bridge two membranes; the hydrophobic tails can thus slide through the groove between the two membranes. Both ATG2 and VPS13 proteins use this strategy to move lipids. The discovery and functions of ATG2 in autophagy and VPS13 proteins are reviewed elsewhere in this Special Issue [5,6].

**Fig. 1:**
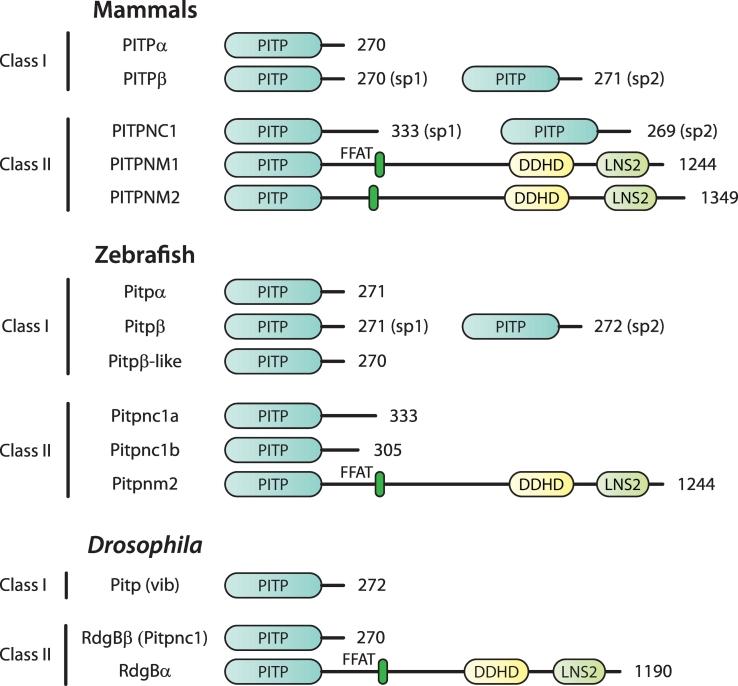
Classification and domain organisation of PITP proteins found in mammals, zebrafish and *Drosophila*. PITP proteins are classified as Class I and II. Class I PITPs bind PI and PC whilst Class II PITPs bind PI and PA. The transcriptional variants of PITPβ and PITPNC1 are indicated alongside where known. In zebrafish, Pitpnc1 is expressed from two separate genes present on different chromosomes.

The family of LTPs, that can bind and transport PI are the phosphatidylinositol transfer proteins, PITPs (Fig. 1). The PITP family in mammalian cells comprises of five members, subdivided into two classes; Class I PITPs, PITPα and PITPβ which are single domain PITPs and three Class II PITPs which are subdivided into Class IIA and Class IIB. Class IIA PITPs are larger proteins containing additional domains that are reviewed elsewhere in this Special Issue [7]. The single Class IIB PITP, PITPNC1 contains a PITP domain with a C-terminal extension of 60 amino acids. This review will be focussed on the three soluble PITPs, PITPα, PITPβ and PITPNC1. Whilst the lipid binding and transfer activities are well-established for these PITPs, the ***cellular*** function of different members of the PITP family are only just beginning to be understood, facilitated by the use of model organisms. New studies reveal how the activity of PITP proteins is harnessed by different cell types to execute multiple functions including phototransduction, sensory behaviour, trafficking from the Golgi and also cell hijacking by viruses. In all these cases, phosphorylated PIs such as PI4P and PI(4,5)P_2_ are the primary mediators. In this review, how specificity of function could be derived based on the lipid-binding properties of the different PITP family members will be discussed.

### 1.1 Introduction to PITPs

The first PITP was identified due to its ability to bind and transfer phosphatidylinositol (PI), hence its name [8]. It was purified from bovine brain and the protein was found to consist of two forms in equal amounts, one form was bound to PI and the second form was bound to phosphatidylcholine (PC). A second PITP was subsequently identified by complementation of a temperature-sensitive Sec14 mutant in *Saccharomyces cerevisiae* [9]. It was simultaneously purified from bovine brain and designated as PITPβ with the original PITP referred to as PITPα [10]. PITPs are found not only in multicellular organisms but also in single cell organisms such as Giardia, *E*. *cuniculi*, *Plasmodium falciparum*, *Dictyostelium* and red algae, indicating their ancient origins [11,12]. PITPs belong to the larger StART family of proteins. However, the absence of PITP proteins in some well-studied model organisms including *Saccharomyces cerevisiae*, *Schizosaccharomyces pombe* and *Arabidopsis thaliana* is noteworthy. These organisms do contain PI transfer activity but this is due to another Class of proteins, the Sec14 family of proteins and are referred to as Sec14-like PITPs (See review by Griac et al. [13] in this Special Issue). PITPs of the StART family and Sec14-like PITPs have no sequence or structural homology [14]. Although members of both families can bind PI in a hydrophobic pocket, the orientation of the bound lipid is different. In PITPs, the headgroup of PI is buried within the pocket with the acyl chains pointing outwards, whilst in Sec14p, the tails are buried and the lipid headgroup is accessible [14]. It is of note that Sec14-like PITPs and StART-like PITPs can be simultaneously present in a simple organism such as *Dictyostelium discoideum* suggesting that the fundamental lipid transfer function of these proteins is specifically harnessed in different biological processes [15].

The StART family belongs to a large superfamily of proteins with a Bet v1 fold, an ancient versatile scaffold for binding hydrophobic ligands [16]. The distinctive arrangement of the secondary structure elements of α-helices and β-sheets gives rise to a three-dimensional structure consisting of a hydrophobic cavity, which is open to the exterior. Proteins with the Bet v1 fold are found among all kingdoms and diversified into numerous families with low sequence similarity but with a common fold during evolution. A large number of proteins make use of this scaffold to bind diverse hydrophobic ligands including phospholipids, ceramide and cholesterol. Both the PITP domain-containing proteins and the StART domain-containing proteins are included in this superfamily [17]. StART family proteins includes CERT that binds ceramide, StaR and MLN64 that binds cholesterol and PCTP that binds PC. More recently, the GRAM domain containing proteins, GramD1a, GramD1b and GramD1c were identified as proteins with a StART domain that binds cholesterol [17,18]. To date only the PITP family of this superfamily with a Bet v1 fold has been shown to bind PI.

The five proteins with a PITP domain present in the human genome are subdivided into two Classes based on two parameters; amino acid sequence and their lipid binding properties [[19], [20], [21]]. Class I PITPs, PITPα and PITPβ, bind and transfer PI and PC whilst Class II PITPs that comprise of PITPNC1, PITPNM1 and PITPNM2 bind and transfer PI and PA. PITPNM proteins are also known as Nir proteins, a nomenclature based on their binding to the N-terminal domain of protein kinase PYK2 (PYK2 N-terminal domain-interacting receptors) [22]. The PITPNM nomenclature will be used here. PITPNM proteins are the mammalian counterparts of RdgBα, first identified in *Drosophila* as a retinal degeneration mutant, and hence its name [23]. The RdgB nomenclature is also used to describe the PITP family. Thus, PITPNC1 is also referred to as RdgBβ [[24], [25], [26]].

**Fig. 2:**
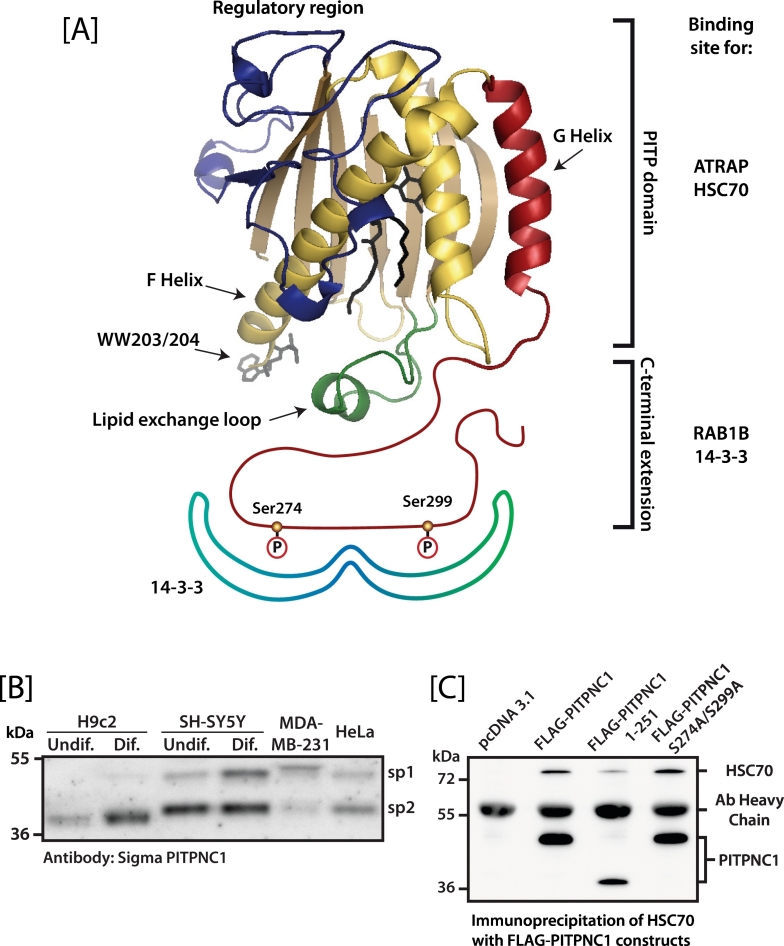
Binding partners of PITPNC1. [A] The modelled structure of PITPNC1-sp1 bound to a 14-3-3 dimer. The lipid in the binding pocket is phosphatidylinositol. The phosphorylation sites, Ser274 and Ser299 which form the binding site for 14-3-3 are indicated. The PITP domain of PITPNC1 was modelled on the template with the PDB code 1T27 using MODELLER (adapted from [26]). The C-terminal extension coloured red is hypothetical. The putative binding sites for RAB1B, HSP70 and ATRAP are indicated. HSP70 and ATRAP bind to the PITP domain whilst 14-3-3 and RAB1B binds to the C-terminal extension. [B] Expression of PITPNC1 splice variants in multiple cell-lines. PITPNC1-sp1 is the longer form at 333 amino acids which contains an extended C-terminus which is disordered. PITPNC1-sp2 is 269 amino acids has a shorter C-terminal extension and does not contain the residues for 14-3-3 binding. H9c2 cells and SH-SY5Y cells were differentiated with all-*trans* retinoic acid in tissue culture media containing 1% fetal calf serum for eight and five days respectively. [C] PITPNC1 binds to HSC70. FLAG-tagged PITPNC1 wild type, a truncated mutant containing residues 1-251 and a mutant with the 14-3-3 binding sites disrupted were transfected into COS-7 cells. All three constructs bind to HSC70.

Although the PITP family comprises of five members in mammals, the presence of splice variants increases their number. PITPα and PITPβ are small compact soluble proteins of ~270 amino acids and share 77% sequence identity and 95% sequence similarity (Fig. 1). In humans, PITPα and PITPβ are localised on chromosome 17p13.3 and 22q12.1 respectively. The major difference between the two PITPs is the C-terminal 20 amino acids. In PITPα and β the C-terminus ends with a helix (G helix in Fig. 2A) followed by an unstructured tail of 11 amino acids. Both proteins exhibit PI and PC binding and transfer activity. A notable difference between the two PITPs is that PITPβ has a higher affinity for binding to membranes and an increased transfer rate [27]. PITPβ is expressed as two splice variants which also differ in their extreme C termini [28]. The C-terminal 15 amino acids of the PITPβ splice variant 1 (sp1) are replaced by an alternative C-terminus of 16 amino acids in PITPβ-sp2. The C-terminus of PITPβ-sp1 is constitutively phosphorylated at Ser262, a residue that is lacking in PITPβ-sp2. PITPβ is highly enriched in liver and the single gene gives rise to seven distinct protein species that differ in their phosphorylation status and the bound lipid state [28]. *In vitro* analysis of the lipid binding properties of the two splice variants suggest minor differences in their biochemical activity. Both variants bind PI and PC and are expressed in most cell types examined. In liver, similar amounts of the two splice variants are expressed at the protein level. PITPβ localises to the Golgi and it was suggested that Golgi localisation was dependent on phosphorylation of Ser262 [29]. However, subsequent studies indicated that both splice variants localise to the Golgi suggesting that phosphorylation is not essential [28,30].

The Class IIB protein, PITPNC1 comprises a PITP domain followed by an unstructured C-terminal extension of 60 amino acids. In mammals there is a single gene that codes for two splice variants [25]. In humans, the *PITPNC1* gene is on chromosome 17. The splice variant 1 is longer with 332 amino acids whilst PITPNC1-sp2 is 268 amino acids. The C-terminal extension in PITPNC1-sp1 contains two serine residues (Ser274 and Ser299) which form binding sites for 14-3-3 proteins when phosphorylated. The shorter splice variant does not interact with 14-3-3 proteins. Compared to Class I PITPs which are expressed in unicellular and multicellular organisms, expression of PITPNC1 is restricted. It is present in mammals, flies, amphibians and fish but not in *C. elegans*. In zebrafish, two separate genes encode for Pitpnc1 proteins, one on chromosome 3 (Pitpnc1a) and the other on chromosome 16 (Pitpnc1b). Pitpnc1a is 331 amino acids long with 81% identity and 90% similarity to PITPNC1-sp1. This protein also binds 14-3-3 proteins. Pitpnc1b is shorter with 305 amino acids and is similar to PITPNC1-sp2 (Fig. 1). The complex expression patterns and isoform splicing of the PITP family across multiple organisms suggests that the proteins have evolved to play discrete roles in specific tissues. In order to understand these roles, it is important to think how the proteins perform their functions. Many studies have been carried out to elucidate the structure of PITPs and how they are able to bind and transfer lipid monomers.

### 1.2 Structure of the PITP domain

Crystal structures of the lipid-loaded and apo-structures of Class I PITPs as well as molecular dynamics simulations have begun to shed light on how PITPs interacts with membranes for lipid exchange [12,[31], [32], [33], [34]]. X-ray crystal structures of three distinct forms of PITPα have been reported; PITPα bound to PC [12] or to PI [32], and one that is devoid of lipid [31]. In addition, the structure of PITPβ loaded with PC is also available [33]. The lipid-loaded structures of either PITPα or PITPβ, regardless of the lipid-bound, are near identical; this excludes conformational changes in the structure regulated by the identity of the encapsulated lipid. The structure of PITP can be subdivided into four regions, the lipid binding core (coloured yellow), the regulatory region (coloured blue), the C-terminal G-helix ending with an unstructured extension (coloured red) and the lipid exchange loop (coloured green) **(**Fig. 2A**)**. Fig. 2A is a modelled structure of PITPNC1 loaded with PI based on the crystal structures and looks remarkably similar to the structures of PITPα/β. The β-strands form a large concave sheet flanked by two long α-helices (coloured yellow in Fig. 2A), that form the lipid binding cavity. The hydrophobic cavity can be occupied by only a single phospholipid molecule. In the case of PITPNC1, it would be either PI or PA. Access to the cavity is obstructed by a ‘lid’ composed of the G-helix and C-terminal unstructured extension (coloured red in Fig. 2A). The headgroup of the bound lipid is accommodated deep inside the cavity whilst the *sn*1 and *sn*2 fatty acyl chains point towards the ‘lid’. With the ‘lid’ closed, the phospholipid is completely enclosed within the protein [35]**.** Due to the location of the inositol ring which is buried deep within the lipid binding cavity, phosphorylation by lipid kinases is not physically possible. In the structure of the lipid-free form, the lipid exchange loop and the G-helix has swung outwards from the main structure, allowing passage of the phospholipid into or out of the cavity.

What controls the dynamics of the protein cycling between the ‘open’ and ‘closed’ is the interaction with a membrane [34,35]. Various regions of the PITPα molecule have been identified that participate in membrane association. This includes the lipid exchange loop, the G-helix and two tryptophan residues (WW203/204) located at the tip of the loop between a β-strand and α-helix F) (see Fig. 2A). Mutation of these residues in PITPβ disrupts Golgi localisation, and *in vitro*, the lipid transfer activity of both PITPα, PITPβ and the PITP domain of *Drosophila* RdgBα is reduced. [32,35,36]. Molecular dynamics simulations of PITPα suggest that the lipid exchange loop inserts into the bilayer upon membrane binding [34]. The most likely amino acid within this loop to have membrane interaction properties is Phe72, however, mutation of this residue to alanine has no impact on lipid binding, lipid transfer or its ability to support phospholipase C signalling in permeabilised cells [32].

Class I and Class II PITPs show differences in their lipid binding characteristics. Both Class I and Class II PITPs can bind and transfer PI; the counter ligand is different. Class I can bind and transfer PC whilst Class II can bind and transfer PA. For PI binding and transfer, four amino acid residues, Thr59, Lys61, Glu86 and Asn90 located on two of the β-strands provide the binding sites for the inositol ring. Mutations of any of those individual residues leads to loss of PI binding but not that of PC in Class I PITPs [32,35,37]. These residues are conserved in the majority of PITP sequences available in the databases, a hallmark that is predictive of PI binding. For PC binding, Cys95 and Phe225 are important. These residues are conserved in Class I but not Class II PITPs, reinforcing their inability to transfer PC [11]. Cys95 is located in the lipid binding cavity and mutation to either threonine or alanine results in loss of PC binding and transfer [37]. In Class II PITPs, this residue is replaced with threonine. Phe225 is not entirely conserved between human and rodents although among species, the amino acid sequences of PITPα and PITPβ are highly conserved (98–99% identity). Whilst Phe225 is conserved in human PITPα and β, and in PITPα in rodents, PITPβ in rodents has a leucine in that position. PC transfer activity is increased when the leucine is mutated to phenylalanine, confirming the importance of this residue for PC transfer. Another feature are four amino acid residues (Gln22, Thr97, Thr114, and Lys195) that are conserved in the majority of PITPs; these residues are important for binding the phosphodiester moiety of either PI or PC.

Residues involved in PA binding remain to be identified in Class II PITPs. The best characterised PA transfer protein is a complex of PRELID1-TRIAP1 which transfers PA from the outer mitochondrial membrane to the inner mitochondrial membrane. Despite no significant sequence homology, the architecture of the PRELID1 domain is reminiscent of the PITP structure, an internal lipid binding pocket surrounded by a β-sheet and α-helices [38,39]. The TRIAP1 domain is helical and is the equivalent of the G-helix in PITPs. Residues important for PA transfer has been identified based on structural analysis and also unbiased genetic approaches in yeast. However, lack of sequence homology between these proteins does not provide any clues about specific amino acid residues required for PA binding in PITPs. Thus, for Class II PITPs, structures with PA bound are needed. This would allow further analysis of the importance of PA binding in phospholipase C signalling and in other functions that these proteins participate in.

### 1.3 The primary function of the PITP domain is to maintain phosphoinositide levels

The recurring theme in PITP protein biology is the ability to maintain phosphoinositide levels [40,41]. Initial studies undertaken in permeabilised cells provided the first clues [[42], [43], [44], [45]]. Depletion of PITPs by permeabilization led to loss of phospholipase C signalling, PI3K signalling, exocytosis and biogenesis of secretory granules and vesicles from the Trans Golgi Network (TGN) [[42], [43], [44], [45], [46], [47], [48], [49]]. From these early studies, a clear concept emerged. PITP proteins promote the synthesis of phosphorylated derivatives of PI including PI4P, PI(4,5)P_2_ and PI(3,4,5)P_3_ (reviewed in [41,50]).

The precise mechanism by which PITPs maintain phosphoinositide levels remains a question. Lipid delivery together with a cofactor function has been proposed [51]. The regulatory mechanism(s) that determine how PITPs are involved in maintaining phosphoinositides levels in different membrane compartments is not known. To date binding partners for Class I PITPs have not been identified. Below we discuss how individual PITPs function in cells at the level of the organism. Studies undertaken in mice, flies, zebrafish and dogs all point to both specific and over-lapping functions of the different members of the PITP families. This is not surprising because phosphoinositides, particularly PI4P and PI(4,5)P_2_, play critical roles in cell signalling, membrane trafficking, cytoskeletal dynamics and cytokinesis [[52], [53], [54], [55]].

Phosphatidylinositol is a negatively charged phospholipid and is present in mammalian cells at around 5–8% depending on cell type [1]. In a typical cell, 5% of the PI will be found as PI4P whilst another 5% will be present as PI(4,5)P_2_. Consumption of PI(4,5)P_2_ during phospholipase C signalling can deplete PI levels by as much as 30% during intensive signalling [56]. PI synthesis is confined to the ER whilst the lipid kinases, PI4KIIIα and PI4P 5-kinases are localised at the plasma membrane; thus, substrate delivery by PITPs is critical to maintain cellular homeostasis. The level of PI at the plasma membrane is negligible and is dependent on delivery from other sources [57,58]. Recent studies suggest that the Golgi, enriched in PI4P, can also contribute to PI(4,5)P_2_ recovery during agonist stimulation [59]. However, how PI4P would be delivered to the plasma membrane was not elucidated. Regardless, PITPs would still be required to deliver PI from the ER to the Golgi. Early studies in rat liver identified the principle organelles that were enriched with PI 4-kinase activity. These were the Golgi and plasma membrane with some activity in the lysosomes [60]. The ER, where PI is synthesised, was devoid of activity as was mitochondria. Four PI 4-kinases divided into Type II and Type III have now been identified in the human genome that are structurally distinct [61,62]. They are expressed in most cells; PI4KIIIβ localises at the Golgi compartment whilst PI4KIIIα localises to the plasma membrane. The Type II PI 4-kinases, PI4KIIα and PI4KIIβ are palmitoylated enzymes and are also found at the Golgi and at endosomes [[61], [62], [63], [64]].

## 2 Class I PITPs

### 2.1 PITPα and PITPβ – neuronal studies in mice and Drosophila

PITPα and PITPβ are the best studied of mammalian PITPs. Both are ubiquitously expressed proteins with PITPα expression being higher compared to PITPβ in most tissues with the exception of liver, lung and neutrophils. The highest levels of PITPα is found in the brain and histochemical analysis in the adult mouse cerebellum shows strong expression in the molecular layer and the granule cell layer [65]. The molecular layer is rich in synaptic contacts due to the dendritic tree of Purkinje cells and the parallel fibres of the granule cell layer. The dendritic spines of the Purkinje cells receive synaptic input from the parallel fibre. (In *Drosophila,* the single Class I PITP is required for dendritic morphology; in the absence of PITP, a reduction in branching is observed [66].) PITPα is also strongly expressed in the hippocampus including CA3 cells [65]. In mice, an essential requirement for PITPα for nervous system development and function was identified in the spontaneously-occurring *vibrator* mutation, which causes neurodegeneration due to reduced expression of PITPα by nearly 80% [67]. The mice show a progressive whole-body tremor and spinocerebellar neurodegeneration. Expression of PITPβ is unperturbed implying a specific function of PITPα or insufficient amounts of PITP proteins. Mice where the PITPα gene is knocked out die within days after birth. These mice show a more complex phenotype; spinocerebellar neurodegeneration, intestinal and hepatic steatosis and hypoglycaemia [68]. Interestingly, when fatty liver disease is induced in zebrafish using perfluorooctane sulfonate, a widely used chemical or a fatty liver mutant, *trappc11*^−/−^ (transport protein particle complex 11), transcription of PITPα is increased [69]. This increase could be a response by the cells trying to counteract the insult. Graded deletion of PITPα indicates that the brain is most affected when PITPα is partially reduced whilst other phenotypes only occur after total reduction in PITPα [70]. The binding of PI to PITPα is essential as a mutant unable to bind PI exhibit the same phenotype as the PITPα-null mice [70].

Previous studies to make *Pitpnb*-null mice or null embryonic stem cells were not successful, concluding that PITPβ was an essential protein [71]. A more recent study was successful in making *Pitpnb*-null mice; they appear to be normal and fertile [72]. Attempts to make mice that were null for both PITP genes was unsuccessful indicating lethality. Instead, both PITPs were deleted specifically from the dorsal forebrain. Mice are born but lack the dorsal forebrain revealing that Class I PITPs are required for the development of the mammalian neocortex [72]. To identify the cellular mechanism leading to this phenotype, both PITPs were deleted in neural stem cells and this resulted in loss of polarity. The stem cells in the developing neocortex are unique bipolar epithelial cells, extending an apical process to the ventricle and a basal process to the basal lamina. In these cells, the Golgi apparatus is confined to the apical process. In the PITP-null cells, the Golgi redistributes from the apical process to a perinuclear location with defects in radial polarity. These phenotypes could be rescued with wild-type PITPα but not a mutant PITP that could not bind and transfer PI. GOLPH3 (Golgi phosphoprotein 3) localises to the Golgi by binding to PI4P and its localisation was disrupted in the PITP-null cells. CERT and FAPP1 also depend on PI4P for their Golgi localisation. Although their localisation was not examined in the PITP-null cells, knockdown of either GOLPH3, CERT or FAPP1 also resulted in Golgi positioning and radial alignment defects. However, only the localisation of GOLPH3 and CERT was dependent on PI4P, that of FAPP1 was not. GOLPH3 recruits MYO18A to regulate Golgi shape and was also found to show Golgi positioning and radial alignment defects when knocked down in neural stem cells of mouse embryos. Thus, PITPα and PITPβ together maintain a Golgi pool of PI4P which is required for Golgi positioning in the neural stem cells.

A recent study in mice where both PITPα and PITPβ were ablated suggests that PITP is also required for the noncanonical planar cell polarity (ncPCP) pathway, the core of which comprises the Wnt-Frizzled/VANGL/PCP network [73]. The membrane traffic of VANGL2 from the TGN to the plasma membrane is disrupted in the absence of both PITPs. During embryonic development, the mammalian cortex expands in a lateral dimension increasing the neocortical area whilst restricting expansion in the radial dimension so that neocortical thickness is restricted, a process known as convergent expansion. Neural stem cells are responsible for this development. During neurogenesis, the nuclei change positions along the apical-basal axis, a process called interkinetic nuclear migration (INM) during the cell cycle to promote convergent extension [74]. At G1, the nuclei undergo apical to basal migration and after completion of S-phase, the nuclei undergo basal to apical migration. Mitosis is confined to the ventricular surface, as the apical plasma membrane harbours the primary cilium that is nucleated by the centrosomes that also build the mitotic spindle [[75], [76], [77]]. INM plays a critical role in cell fate determination and is compromised in neural stem cells when both Class I PITPs are deleted. This results in a thickened neocortex and perturbed curvature of its ventricular surface. These phenotypes are recapitulated in mouse embryos individually ablated for VANGL2 and Celsr1 suggesting that the ncPCP signalling is somehow connected to PITPs. PITPs appear to be required for the membrane trafficking of VANGL2 from the TGN to the neural stem cell surface. GOLPH3 is not required for this process suggesting that different PI4P effectors may be involved. These results suggest that neural development requires PITP for membrane trafficking of specific cargo proteins. These results can be best understood if the function of the Class I PITPs was to maintain Golgi PI4P. Loss of GOLPH3, CERT, and FAPP1 all depend on PI4P and moreover, PI4P participates in vesicle biogenesis from the TGN. These new studies provide further support that the PITPs are important for maintaining phosphoinositide levels in the Golgi compartment.

The importance of PITP in neuronal function is further supported by studies in *Drosophila* neural stem cells or neuroblasts, where the single Class I PITP (also known as *vib or gio*) and the PI 4-kinase, PI4KIIIα were found to govern neuroblast polarity by anchoring non-muscle myosin II [78]. Neuroblasts divide asymmetrically to generate a self-renewing neuroblast and a differentiating daughter cell. During asymmetric division, polarized distribution of proteins occurs and are asymmetrically segregated into daughter cells. In PITP mutants, neuroblast homeostasis was perturbed with loss of neuroblasts. In addition, PITP was required for the localisation of apical and basal proteins and their faithful segregation in dividing neuroblasts. For example, in PITP mutants, aPKC and Par6 were found to redistribute from the apical cortex to the cytoplasm.

The non-muscle myosin II regulatory light chain protein, Spaghetti-squash (Sqh) regulates asymmetric localisation of basal cell fate determinants in dividing neuroblasts. In Sqh mutants, aPKC is also mislocalised similarly to that seen in PITP mutant cells. Normally, Sqh is partially cytoplasmic with some localisation at the cell cortex in interphase cells and during metaphase it localises uniformly to the cell cortex. In PITP mutant cells, Sqh is completely cytosolic at interphase and partially cytosolic at metaphase indicating that Sqh is targeted to the cortex *via* PITP. PITP is responsible for promoting the synthesis of PI4P, and localisation of PI4P at the plasma membrane is lost in PITP mutants. Of the three PI 4-kinases expressed in *Drosophila*, PI4KIIIα was required for both neuroblast homeostasis and for asymmetric division. It was found to co-localise with Sqh to the cell cortex. Thus, PITP recruits Sqh and a direct interaction between the two proteins was demonstrated. In addition, Sqh also binds PI4P. Surprisingly, when Sqh was mutated, PI4P localisation was disrupted. Thus, Sqh requires both PITP and PI4P for recruitment to the cell cortex and PI4KIIIα provides the PI4P whilst PITP provides PI for conversion to PI4P. PITP^T59E^ mutant is a well-characterised mutant; it is defective in PI binding and transfer but not that of PC. The equivalent mutant in *Drosophila* is PITP^T63E^ and this mutant was unable to localise Sqh to the cell cortex. This comprehensive analysis provides the first evidence of how the complex interplay between PITP, PI4PKIIIα, and PI4P contribute to the recruitment of Sqh. Both PITP and Sqh are required for plasma membrane production of PI4P.

These two studies illustrate how PITPs contribute to localised PI4P production [78,79]. In the mouse model, where both Class I PITPs were disrupted, polarity of neural stem cells is lost. This is due to loss of PI4P at the Golgi where it is required for the trafficking of VANGL2 from the TGN to the neural stem cell surface. In the *Drosophila* model, the single PITP is required for PI4P production at the plasma membrane for recruitment of the non-muscle myosin II (Sqh) that governs neuroblast polarity. In these two studies, PITPs provide PI to different PI4Ks at separate localisations; PI4P at the plasma membrane through PI4KIIIα and at the Golgi through PI4KIIIβ. This rules out direct interactions between PITPs and PI4Ks.

### 2.2 GOLPH3, RABs and PITPs

*Drosophila* PITP is also required for cytokinesis in spermatocytes and larval neuroblasts [80,81]. In addition to PITP, the PI4KIIIβ (*Fwd*), GOLPH3 and Rab11 are also required for cytokinesis [[82], [83], [84], [85]]. GOLPH3 is a highly conserved protein and binds to PI4P with high affinity [86]. It is essential for contractile ring formation and Rab11 localisation to the cleavage furrow during cytokinesis [87]. GOLPH3 binds to Sqh, non-muscle myosin II light chain required for actomyosin contraction at the cleavage site. PITP and the PI4KIIIβ participate in PI4P synthesis and are also required for the actomyosin ring constriction and cleavage furrow ingression. For cleavage furrow ingression, Golgi-derived vesicles are required and mutations in PITP or PI4KIIIβ results in the abnormal localisation of Golgi-derived vesicles at the cell equator. Failure of fusion of these Golgi-derived vesicles with the invaginating furrow is responsible for the defects in cytokinesis. The Golgi-derived vesicles are Rab11-positive and are responsible for PITP and PI4KIIIβ recruitment. The vesicles are recruited to the cleavage furrow and this requires the exocyst complex [88]. Thus PITP, together with Rab11 and PI4KIIIβ, is essential during exocyst-dependent membrane addition during cytokinesis in spermatocytes [81,84,88]. The single *Drosophila* PITP can participate in PI4P generation using different PI 4-kinases at different locations. During cytokinesis, PITP provides PI to PI4KIIIβ whilst during asymmetric division of neuroblasts, PITP collaborates with PI4KIIIα.

In mammalian cells, GOLPH3 is localised to the TGN. Several studies suggest that it regulates anterograde trafficking of vesicles from the TGN to the plasma membrane. Other studies suggest that GOLPH3 is required for recycling glycosylation enzymes from the TGN to the *cis*-Golgi in COP1-coated vesicles [[89], [90], [91], [92]]. GOLPH3 is over-expressed in many cancers and it is proposed that GOLPH3 might contribute to cellular transformation by affecting glycosylation of key cancer-relevant glyco-proteins [90,93,94]. GOLPH3 also regulates Golgi to plasma membrane trafficking and contributes to malignant secretory phenotypes. In this case, another PITP, PITPNC1 (which will be described later), recruits RAB1B to the Golgi which interacts with GOLPH3 to recruit MYO18A. MYO18A by connecting to actin filaments, is thought to stretch the Golgi to cause membrane curvature and allow vesicle budding from the Golgi [86,95]. PI4P at the Golgi is synthesised by PI4KIIIβ and this kinase is also amplified in cancer [97]. Thus, the PITPNC1/RAB1B/GOLPH3/MYO18A/F-actin module participates in anterograde traffic and in some cancers, allows malignant secretion to take place. Whether PITPNC1 is required for intra-Golgi retrograde traffic is not known, but it is interesting to note that PITPβ is required for COP1-mediated traffic from the Golgi to the ER at least in HeLa cells [37]. Mutational analysis indicates that PITP has to be competent for both PI and PC exchange and also competent to dock on membranes [37]. Studies in both *Drosophila* and mammalian cells suggest a collaboration between RABs, GOLPH3 and PITPs as a recurring theme. The importance of PI4P in the recruitment of GOLPH3 is well-established but the link between RABs and GOLPH3 remains to be validated.

### 2.3 PITPα and Duchenne muscular dystrophy

Duchenne muscular dystrophy (DMD), an X-linked degenerative muscle disorder, is caused by the absence of a functional dystrophin protein. Dystrophin is a rod-shaped protein that links the intracellular cytoskeleton network to an integral membrane glycoprotein at the sarcolemma to provide structural stability and a signalling anchor to the muscle fibres. Loss of dystrophin causes a gradual decline of muscle function due to muscle degeneration. Recent studies in a colony of golden retriever muscular dystrophy dogs have identified that animals with decreased expression of PITPα exhibit a very mild phenotype [98]. In contrast, dogs that exhibit a severe phenotype have higher levels of PITPα compared to normal dogs. In these dogs, decreased expression of PITPα was associated with increased phosphorylated Akt (pAkt) expression and decreased PTEN (phosphatase and tensin homolog) levels. Further studies in DMD-patient derived muscle cells and in dystrophin-deficient zebrafish supported these observations. PITPα downregulation in DMD patient-derived muscle cells enhanced the formation of mature myotubes and increased pAkt [98]. PITPα knockdown by injection of morpholino oligonucleotides in a dystrophin-deficient zebrafish also increased pAkt, rescued the abnormal muscle phenotype, and improved long-term survival.

Using the zebrafish dystrophin-deficient model, an inhibitor of the phosphodiesterase 10A (PDE10A) was found to reduce the manifestation of the dystrophic muscle phenotype. Moreover, PDE10A inhibition in zebrafish and in DMD patient-derived myoblasts were also associated with reduction of PITPα expression [99]. Thus, these results indicate a reduction in PITPα as a protective genetic modifier in dystrophin-deficient model systems and permits the escape of the dystrophic phenotype. Decreased PITPα expression was associated with decreased levels of the PTEN and with increased Akt-phosphorylation. These effects promote muscle growth and metabolism to counteract the pathologies associated with muscular dystrophy. Similar changes in PTEN and Akt regulation were also observed following shRNA-mediated knockdown of PITPα in human DMD myoblasts and upon PITPα knockdown in a dystrophin-deficient sapje mutant zebrafish DMD model.

How does loss of PITPα lead to a decrease in PTEN with increased pAkt? A plausible explanation could be the regulation of PTEN by PI(4,5)P_2_. PTEN binds to PI(4,5)P_2_ allowing recruitment to the membrane for synthesis of PI(3,4,5)P_3_ [[100], [101], [102]]. In the absence of PITPα, depletion of a specific pool of PI(4,5)P_2_ utilised by PTEN for its recruitment, would result in increased PI(3,4,5)P_3_ and thus pAkt. Why does inhibition of PDE10A cause a decrease in PITPα? PDE10A can hydrolyse both cAMP and cGMP. Cyclic nucleotides could regulate PITPα levels through transcriptional regulation and this link needs further investigation. Regardless, these are interesting developments that could result in novel approaches to ameliorate the phenotypes in Muscular Duchenne patients.

### 2.4 A requirement for PITPs in megakaryocytes and platelet function

Mice knockouts of Class I PITPα results in severe phenotypes and thus conditional knockouts in specific cell-types would provide a better understanding of PITPα function. Conditional knockout mice lacking PITPα or both PITPα and PITPβ reveal important roles for PITPs in megakaryocytes and in platelets. Megakaryotes are derived from pluripotent hematopoietic stem cells (HSCs) and are large cells of 100 μm that give rise to platelets. Platelets are small cells (2-3 μm) with no nucleus and are important for haemostasis. Platelets and megakaryotes are characterised by the presence of α-granules and dense granules that contain a variety of mediators. α-Granules contain proteins essential for platelet adhesion during vascular repair such as fibrinogen and Von Willebrand Factor (VWF), P-selectin, and angiogenesis regulatory proteins. Dense granules function predominantly to recruit additional platelets to sites of vascular damage and secrete ADP and ATP. ADP is a weak agonist triggering platelet shape change, granule release and aggregation.

Megakaryotes lacking both Class I PITPs have a defect in α-granule morphology and show increased secretion of its contents including VWF, thrombospondin 1 and TGFβ1 into the extracellular medium. TGFβ1 maintains HSC quiescence and this results in decreased haematopoiesis. These data indicate that PITPs have a role in membrane trafficking, and in particular, in the biogenesis of secretory α-granules. No defects were observed in dense granules. This is not surprising as the biogenesis of α-granules and dense granules are distinct. In megakaryocytes, the biogenesis of α-granules is formed from the TGN and early endosomes which then mature in multivesicular bodies (MVBs) [103]. Thus, the proteins stored in α-granule are acquired from both the ER-to-TGN biosynthetic pathway and from the endocytic pathway that brings in plasma proteins. The α-granules and the MVBs from the double PITP knockout megakaryocytes are less electron-dense and it was suggested that the α-granule constituents were misdirected towards constitutive secretion into the medium [104]. Knockout of the single PITPα resulted in a milder phenotype. At what step PITPs are required for α-granule biogenesis remains to be determined. PI4P has several roles in transport from the TGN, suggesting that it is the prime candidate [55]. PI4P can participate in the recruitment of coat proteins for vesicle biogenesis. Thus, PITPs are likely to provide PI for phosphorylation by the organelle-resident PI 4-kinases. The TGN and endosomes are both endowed with different PI4Ks and would imply localised production of PI4P at these sites.

Conditional knockouts of PITPα from mouse platelets reveals a requirement in thrombin-stimulated phospholipase C signalling. A reduction in IP_3_ production, in cytosolic Ca^2+^ and in PI4P and PI(4,5)P_2_ levels suggest defects in substrate provision for phospholipase C activity upon thrombin addition [105]. Nonetheless, some functional responses such as aggregation and α-granule release are near normal when platelets are stimulated with thrombin. Although the major physiological function of platelets is in haemostasis, they can also contribute to tumour metastasis [[106], [107], [108]]. Thus, mice, with PITPα missing in their platelets, develop fewer lung metastases compared to normal mice when challenged with tumour cells. Normal platelets adhere to tumour cells forming a shroud and this protects the tumour cells from detection by immune cells. The tumour cells induce deposition of fibrin and thrombin generation; both of these functions are defective in platelets null for PITPα. Thus, mice lacking PITPα in their platelets are protected from metastasis [105]. This very likely results from the failure of the platelets to expose phosphatidylserine (PS) by TMEM16F on the extracellular surface, a process that requires a rise in cytosol Ca^2+^ [109]. Exposed PS is required for thrombin generation and fibrin formation allowing tumour cells protection from the immune system.

### 2.5 PITPβ participation in viral infection

**Fig. 3:**
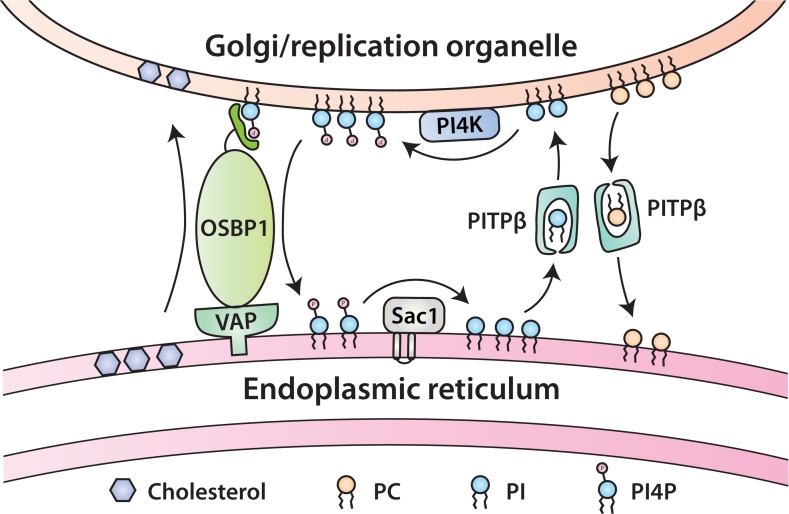
PITPβ participates in cholesterol transport from the ER to the Golgi. PITPβ transfers PI to the Golgi for conversion to PI4P which is then used for the transport of cholesterol from the ER by OSBP1. PI4P is converted back into PI by Sac1. As described in the text and not shown here, during viral replication, OSBP, PITPβ, and Sac1 are used to transport cholesterol to the replication organelle.

PITPβ has been implicated in viral replication of positive-strand RNA viruses of the *Picornaviridae* family. For viral RNA replication, the membranes of the ER and the Golgi are rearranged to generate a specific compartment, the replication organelle. PI4P is produced at the replication organelle by recruitment of PI4K *via* a Golgi-localised protein, ACBD3. PI4P production is commonly observed at viral replication sites for many other viruses [[110], [111], [112]]. Recent studies with the Aichi virus, which causes acute gastroenteritis in humans, hijacks OSBP to transport cholesterol from the ER to the replication organelle. This occurs at the ER-replication organelle membrane contact site. In addition to OSBP, VAPA/B, SAC1 and PITPβ are part of the cholesterol transport pathway. Depletion of any of these proteins results in loss of viral RNA replication. A complex comprising of viral proteins, ACBD3 and PI4KIIIβ synthesises PI4P at the sites for viral RNA replication. OSBP is a transfer protein that uses the PI4P as a counter ligand to transfer cholesterol from the ER to the replication organelle [113,114]. At the ER, PI4P is dephosphorylated to PI by the PI4P phosphatase, SAC1. To regenerate PI4P at the replication organelle, PI is transferred by PITPβ (see Fig. 3). For Aichi virus replication, all the component proteins of the cholesterol transport machinery, OSBP, VAP, SAC1, and PITPβ, are all essential host factors that are directly recruited to the RNA replication organelle [115,116]. Some viruses including SAR2-CoV2 use another PITP, the multi-domain PITPNM1 to accomplish the same function [117,118]. The strategy of combining multiple LTPs, OSBP and PITPβ or PITPNM1 for cholesterol transport is used by other organisms. In *Toxoplasma gondii*, instead of using multiple LTPs, a single protein of nearly 2000 amino acids that contains both the OSBP and the PITP domain can facilitate cholesterol transport [11,26,119].

## 3 Class IIB PITPNC1

The last of the mammalian PITPs to be identified was PITPNC1 (also known as RdgBβ), cloned from human fetal brain. PITPNC1 is longer than PITPα/β; 333 amino acids compared to 270 amino acids [24]. Like Class I PITPs, the 38 kDa protein comprises the PITP domain followed by a C-terminal extension of 60 amino acids that is disordered. The gene is located on chromosome 17q24.2 with a pseudogene located on the long arm of chromosome 1q32.1. Sequence analysis allowed PITPNC1 to be classified with RdgB proteins as a Class IIB PITP [20]. PITPNC1 is ubiquitously expressed with enrichment in heart, muscle, kidney, liver and peripheral blood leukocytes.

The *PITPNC1* gene is alternatively spliced giving rise to two splice variants. Splice variant 1 is the original protein at 333 amino acids; the shorter splice variant, sp2, is 269 amino acids. [25,120]. GFP-fusions of the two splice variants indicate that both proteins localise to the cytosol with the shorter variant also in the nucleus. Using *in situ* hybridization, the gene expression of the shorter form in the mouse brain was confined to the embryonic stage, whilst the longer form was widely expressed in the gray matter of pre- and postnatal brains. The dentate gyrus, the thalamus and the Purkinje cell layer in the cerebellum showed enrichment in the adult brain [120]. Interestingly, the gene expression of the two splice variants was also discrete in other mouse tissues; adult heart expressed both variants, the small intestine expressed the short form whilst the liver, kidney and testis only expressed the larger form.

PITPNC1 orthologues are restricted to jawed vertebrates (*Gnathostomata*) including, mammals, birds, turtles, lizards, amphibians and fish. In zebrafish. Pitpnc1 is expressed as two separate genes on different chromosomes instead of as splice variants. The pitpnc1a gene is present on chromosome 3 and encodes a protein of 331 amino acids, that shows 81% identity to human PITPNC1-sp1. The pitpnc1b gene is present on chromosome 16 and encodes a shorter protein of 305 amino acids which is the equivalent of the human PITPNC1-sp2 protein. The two proteins are differentially expressed: pitpnc1a is exclusively present in the brain with high expression in the forebrain, habenula and cerebellum. Pitpnc1b is excluded from the brain and is expressed in the pharyngeal arches, olfactory vesicles and the pronephric duct. The differential tissue distribution of the two proteins strongly indicate separate functions for these two proteins, likely to extend to the splice variants present in humans.

In cell-lines, both splice variants appear to be present with the expression of the smaller sp2 variant present at higher levels in most cases **(**Fig. 2B**)**. Moreover, expression of both splice variants is increased following differentiation of the H92c cardiomyoblasts and SH-SY5Y neuronal cell-line using all-*trans* retinoic acid [121]. Interestingly, the breast cancer cell line, MDA-MB-231 is an exception which expresses a higher amount of sp1 compared to sp2. The observation that expression of PITPNC1 increases following differentiation indicates that it is required for a cellular process in a mature cell such as a terminally differentiated cardiomyocytes and neurons.

### 3.1 Binding partners for PITPNC1

Several proteins have been identified that can bind PITPNC1. These are 14-3-3 proteins, ATRAP, HSC70 and RAB1B (Fig. 2A). The C-terminus of the longer splice variant, PITPNC1-sp1 contains two serine residues, (Ser274 and Ser299) which are phosphorylated creating a docking site for 14-3-3 proteins [25,26]. Phosphorylation is very likely by protein kinase C based on inhibitor studies [26]. Binding of 14-3-3 masks two PEST (Pro, Glu, Ser, Thr) sequences, that are present in proteins that show rapid turnover. Thus 14-3-3 binding protects the protein from degradation by the proteasome. However, compared to PITPβ, which remains stable for up to 6 h, wild type PITPNC1-sp1 has a half-life of about 4 h, whilst the mutant protein that does not bind 14-3-3 is reduced to 2 h. In addition to binding 14-3-3 proteins, PITPNC1 binds the transmembrane adapter protein, ATRAP. This interaction is dependent on protein kinase C activation. ATRAP can bind to both splice variants identifying the PITP domain as the site of interaction. Other proteins that binds to PITPNC1 are RAB1B [122] and HSC70 (heat shock cognate 70), a molecular chaperone (Blunsom and Cockcroft, unpublished) (Fig. 2C). HSC70 also binds to a truncated form of PITPNC1, one without the C-terminal region present. HSC70 is involved in facilitating protein maturation events and can transiently interact with nascent polypeptides in the process of synthesis. It is also involved in targeting proteins to the ubiquitin/proteasome machinery for ubiquitin-dependent degradation. This is in accord with the short half-life of the protein. RAB1B binding to PITPNC1 is not well-characterised but as binding of RAB1B to PITPNC1 is increased when a mutant that cannot bind 14-3-3 is used, this suggests that the binding site is in the C-terminal region, probably competing with 14-3-3 binding [122].

A study of the lipid binding and transfer properties of PITPNC1 reveals its unusual characteristics when compared to Class I or Class IIA PITPs. The PITP domain shares only 39% identity with Class I and Class IIA proteins. The first notable feature of PITPNC1 is its poor *in vitro* PI transfer activity compared to both Class I and Class 2A PITPs. Secondly, it binds PA with very high affinity. Whilst PITPα can bind equal amount of PI and PC, PITPNC1 binds equal amounts of PI and PA. Considering that PC is generally present at 40% of total phospholipids whilst PA is present at less than 1%, PA binding is of very high affinity. PITPNC1, when purified from *E. coli,* is also preloaded with PA and PG despite the low level of PA present. When PITPNC1 is exposed to permeabilised HL60 cells, the PA species bound to PITPNC1 are enriched for C16:0/C16:1 and C16:1/C18:1, and increased binding to PA is observed at the expense of PI when cellular PA levels are increased. This is particularly the case when phospholipase D is activated. The fatty acid composition of the PA bound to PITPNC1 is characteristic of PC. Thus, PITPNC1 appears to respond to changes in PA levels as can occur during increased activity of phospholipase D or of diacylglycerol kinases. The significance of this is not known.

The biological function of Pitpnc1a has been examined in zebrafish. Pitpnc1a was knocked out using CRISPR and the adult fish developed normally and were fertile. The major phenotype observed in Pitpnc1a-null zebrafish larvae is behavioural. The larvae lacking Pitpnc1a are hyperactive, with increased activity to touch, increased twitching and increase in waking activity. The increased neuronal activity was also evident biochemically; cFos expression was increased in many areas of the brain in the knockout animals. The cause of the hyperactivity is due to upregulation of IGF-1 receptor signalling. IGF-1 signalling is restrained by IGFBP2, and its secretion is likely to be impaired in cells lacking Pitpnc1a. This conclusion is supported by studies in cultured cells lines where knockdown of PITPNC1 result in a reduction of IGFBP2 secretion [123]. A recent study has identified that sleep disturbances can be affected by levels of IGF-1 by modulating the activity of orexin neurons [124]. This further supports that the PITPNC1/IGFBP2/IGF-1-axis can regulate neuronal function and impacts on physiological behaviour.

Phosphatidic acid has emerged as a major regulator of cell function [[125], [126], [127]]. For example, studies in mice reveal that PLD1 is the major source of PA in the hippocampus and knockout mice show deficits in behaviour [128]. PA can also be made from DAG by diacylglycerol kinases (DGKs). Mammals express ten different enzymes that can use DAG with different fatty acid compositions [129,130]. DGKs are known to participate in neuronal function, cancer and Type II diabetes [129]. There is a strong possibility that PITPNC1 could be one of the effector proteins that senses PA to execute downstream function. As described below, PITPNC1 regulates malignant secretion from the Golgi resulting in metastasis.

### 3.2 PITPNC1 and cancer

Cellular lipid metabolism is altered in cancer. There are major changes in the amounts and the fatty acid profiles of the different lipids including PI and PA [131,132]. It is notable that breast cancer cell-lines have increased levels of PA, a lipid that is bound by PITPNC1 with high affinity [132,133]. In addition, the fatty acyl composition of PI is altered such that the acyl chain lengths are reduced [133]. Several studies indicate that PITPNC1 plays a pro-cancer role; it can promote tumour angiogenesis, metastasis, malignant secretion in breast cancer, omental metastasis of gastric cancer and in the development of radio resistance in colorectal cancer. Furthermore, PITPNC1 levels are increased in several different types of cancer (https://Cancer.Sanger.ac.uk/cosmic).

PITPNC1 was first recognised as a biomarker for human breast cancer metastasis [123]. A microRNA (miRNA)-126 that is silenced in a variety of human cancers, regulates the expression of IGFBP2, PITPNC1 and MERTK. (It is of note that miRNA-126 is also downregulated in paediatric B cells precursor acute lymphoblastic leukemia; however, its overexpression in MHH-CALL-3 leukemic cells does not reduce the expression levels of PITPNC1 [134].) The expression of IGFBP2 was dependent on PITPNC1. Subsequent work revealed that PITPNC1 was responsible for malignant secretion from the Golgi by facilitating the recruitment of RAB1B. RAB1B recruits GOLPH3 for driving vesicle formation for secretion of factors that drives invasion and angiogenesis. GOLPH3 is also highly expressed in many cancers. As discussed previously, many studies suggest that GOLPH3 participates in retrograde transport of glycosylation enzymes in COP1-coated vesicles from the TGN to the earlier Golgi compartments [92]. Thus, the mechanism by which PITPNC1 enhances secretion needs further work.

Two recent studies have examined the role of PITPNC1 in gastric cancer omental metastasis [135] and in radio-resistance in colorectal cancer [136]. Gastric cancer cells have elevated levels of PITPNC1 and this is due to the increased levels of cytokines, TNFα and IL6 secreted from adipocytes. Increased expression of PITPNC1 in gastric cancer cells results in increased activity of the transcription factors SREBP1 (Sterol regulatory element-binding protein 1) and PPARγ (Peroxisome proliferator-activated receptor γ), regulating the expression of the fatty acid transporter, CD36 and carnitine O-palmitoyl transferase 1, which transports fatty acids into mitochondria. Effectively, PITPNC1 rewires the metabolism of the cancer cells to utilise fatty acids as fuel allowing the cancer cell to metastasise. What is of note is that the PITPNC1 responsible for this process appears to be the splice variant 2, the shorter form, judging by the size of the protein. The splice variant, sp2, would not be expected to bind RAB1B suggesting that the two splice variants function in cancer metastasis by different mechanisms.

PITPNC1 is also implicated in resistance to radiotherapy used for therapy for colorectal cancer. Patients that show radio-resistance have a significantly higher level of PITPNC1 expression in rectal cancer tissue and produce significantly fewer ROS (reactive oxygen species). It is thought that PITPNC1 negatively regulates ROS production required for radiation-induced cell death. In support of this, PITPNC1 knockdown increased ROS production that could be reversed by a ROS scavenger [136].

In summary, PITPNC1 involvement in cancer is multifactorial. In addition to participating in secretion of malignant factors from the Golgi, in other cancers, the function is different.

### 3.3 PITPNC1 and Type 2 diabetes

GWAS studies had identified an expression-associated SNP in PITPNC1 (rs8866) as a risk factor for Type 2 diabetes [137]. Further support comes from another study where PITPNC1 was also identified as a risk locus using a genome-wide association study by proxy (GWAX) [138]. In GWAX, family members are used in the analysis. Observations that IGF-1 signalling is enhanced when PITPNC1 is deleted suggest that insulin signalling might be similarly affected by PITPNC1 [139].

## 4 Perspectives and future directions

PITPs are ubiquitous proteins and their biochemical activity of lipid transfer makes them well-suited for providing PI for conversion to PI4P and PI(4,5)P_2_. The emerging picture is that PITPs function with different PI4K enzymes. PI4K enzymes are diverse and are localised in multiple organelles including the Golgi and the plasma membrane. Studies using model organisms indicate the diversity in the usage of phosphorylated PIs. This can occur both at the plasma membrane or the Golgi. At the plasma membrane, PITPs are required for PI(4,5)P_2_ production for phospholipase C, and at the Golgi, PI4P is required for membrane traffic. Whilst the importance of PI binding and transfer is well-established in this process, it is not known whether PC metabolism is also affected by PITPs. Only one study has examined whether PC binding is important. In HeLa cells, COP1-dependent retrograde traffic was disrupted in PITPβ-knockdown cells and rescue was only possible with a protein that retained both PI and PC transfer activity [37]. Further work is required to test this in other systems including in model organisms. An outstanding issue is the regulation of the PITP transfer activity. In cells, it is noted that the Class I PITPs are constantly sampling membranes undergoing an ‘open’ and ‘closed’ conformation [35]. Moreover, it is also noted that upon stimulation of cell surface receptors with appropriate agonists, PITPα and PITPβ can dynamically interact with PI and with PC [140]. This dynamic behaviour must be subject to regulatory controls and studies using new techniques such as hydrogen‑deuterium exchange mass spectrometry may provide a deeper understanding of how these proteins interact with membranes.

Although PITPα and PITPβ are very similar is sequence, they appear to have both overlapping as well as specific functions. In both cases, the PITP activity is harnessed to participate in PI4P production. Depending on the compartment where PI4P is required, PI is provided by PITPs to be used by different PI 4-kinases. Thus, at the plasma membrane, PI4KIIIα is responsible for PI4P synthesis whilst at the Golgi PI4KIIIβ is responsible. This implies that PITPs do not directly interact with the PI4Ks but very likely deposit the lipid within the vicinity of the kinase. The interesting question is that of the composition of the membrane surrounding the area of PI4K localisation. Does the composition influence where PI is deposited in the membrane? Parameters that influence lipid transfer *in vitro* include membrane charge, fluidity, lipid composition and curvature [141,142]. How this operates in a cellular context is an unknown and this will require development of new tools to observe lipid transfer in real time in a living cell. However, studies in permeabilised cells and also in intact platelets indirectly indicate that delivery of PI does take place at the plasma membrane to account for increased hydrolysis of PI(4,5)P_2_. Indeed, phospholipase C prefers to utilises the newly-synthesised PI(4,5)P_2_ rather that the resident PI(4,5)P_2_ in the membrane [51,105]. This is not surprising as much of the PI(4,5)P_2_ may be unavailable, due to being bound to other proteins, including the actin cytoskeleton.

Not only is PITPα required for maintaining PLC activity in platelets stimulated by thrombin, it also has functions at the Golgi. In mice, PITPα and β work with PI4KIIIα in polarity establishment during neurogenesis [72]. In *Drosophila,* the single Class I PITP works together with Rab11 and PI4KIIIβ during exocyst-dependent membrane addition during cytokinesis [81,84,88] and also works together with PI4KIIIα in polarity establishment during neurogenesis [78].

In contrast to Class I PITPs, the exact molecular mechanism behind the function of PITPNC1 remains enigmatic. Having said this, recent work in model organisms have begun to unlock the cellular function of PITPNC1. These studies have started to reveal some overlapping functions with Class I PITPs that are of interest to explore further. Broadly, PITPNC1 seems to regulate the morphology and function of the Golgi, and aberrant levels of PITPNC1 can alter levels of traffic through the secretory pathway. An example of this is seen in cancer, where increased levels of PITPNC1 lead to enhanced secretion of factors that can promote metastases of tumour cells [122]. Similarly, Pitpnc1a knockout studies in the zebrafish show that IGFBP2 secretion from neurons is reduced leading to over activation of the IGF-1 axis which manifests in behavioural phenotypes [139]. Although it appears that both Class I and Class II PITPs can regulate Golgi function, it is still unclear whether they achieve this by exactly the same biochemical mechanism.

Whilst PITPNC1 is classified as a lipid transfer protein, it shows poor transfer activity for both PI and PA compared to other PITPs. This observation suggests that the regulation of Golgi function occurs *via* a different mechanism to that achieved by PITPα and PITPβ. One thing to note is that PITPNC1 is able to bind PA with very high affinity and this suggests that PITPNC1 is likely to participate in some aspect of PA signalling. Several studies have shown that PA produced *via* phospholipase D maintains the structural integrity of the Golgi [143]. Additionally, inhibition of PA production alters the structure of the Golgi and inhibits secretion from neuroendocrine cells [[143], [144], [145], [146], [147], [148]]. In particular, mono-saturated PA which preferentially binds to PITPNC1, regulates a number of exocytotic events [148]. More work is required to ascertain if PITPNC1 is regulating these processes through binding and/or transfer of PI/PA. PITPNC1 has been shown to have links to various pathologies including cancer, behavioural abnormalities and diabetes. This makes PITPNC1 a good candidate for further analysis to expand our knowledge of the mechanisms that lead to certain pathologies. These potential studies could lead to new therapeutic avenues for some diseases that have current clinical unmet needs.

The Class I and Class II PITPs are able to bind distinct combinations of phospholipids. Class I PITPs can bind PI and PC and the Class II PITPs can bind PI and PA. The question remains what physiological processes are supported by these specific phospholipid binding profiles. Experiments should be carried out in tissue-relevant cell models, which aim to knock out PITPs and assess the phenotypes induced by these changes. Rescue experiments could then be carried out with specific mutant PITPs with altered lipid binding profiles to pinpoint exact structure/function relationships. One area of focus for these phenotypic assays should be the lipid composition, morphology and secretory function of the Golgi apparatus. This is because the function of this organelle seems to link most phenotypes observed when PITP function is perturbed. It will be important to choose cellular systems that show high expression of the PITPs as pathologies from perturbation of PITP function usually occurs in tissues whereby the expression levels of PITPs are high.

There are four key areas of research that are outstanding. Development of inhibitors that can inhibit specific lipid binding to PITPs, structure of PITPNC1 bound to PA and to 14-3-3 and analysis of the splice variants of PITPβ and of PITPNC1. The splice variants of PITPNC1 is particularly interesting as the zebrafish model organism expresses the two proteins from separate genes. Finally, do soluble PITPs function at membrane contact sites and if so, how are they recruited? In conclusion, there is much to be discovered.

## CRediT authorship contribution statement

This article was jointly written by Tim Ashlin, Nicholas Blunsom and Shamshad Cockcroft.

## Declaration of competing interest

The authors declare no competing interests.
